# Lack of scarring is not always a sign of cardiac health: Functional and molecular characterization of the rat heart’s following chronic reperfusion

**DOI:** 10.1371/journal.pone.0209190

**Published:** 2018-12-20

**Authors:** Ana Carolina Mieko Omoto, Fábio Nelson Gava, Carlos Alberto Aguiar Silva, Hadder Batista Silva, Juliana Montenegro Parente, Rafael Menezes Costa, Michele Mazzaron Castro, Rita de Cássia Tostes, Helio Cesar Salgado, Rubens Fazan

**Affiliations:** 1 Department of Physiology, Ribeirão Preto Medical School, University of São Paulo, Ribeirão Preto, São Paulo, Brazil; 2 Department of Pharmacology, Ribeirão Preto Medical School, University of São Paulo, Ribeirão Preto, São Paulo, Brazil; Max Delbruck Centrum fur Molekulare Medizin Berlin Buch, GERMANY

## Abstract

Even though the coronary reperfusion process is the most important tool to preserve cardiac function, after myocardial infarction, reperfusion of acutely ischemic myocardium can induce injury. We aimed to evaluate the functional and molecular aspects 4 weeks after myocardial ischemia-reperfusion (IR) in rats. Male Wistar rats (N = 47) were subjected to myocardial IR by short-term (30 min) ligation and subsequent reperfusion of the left descending coronary artery. Control rats (N = 7) underwent the same surgical maneuver without coronary ligation. After 4 weeks, rats had their cardiac function examined by ventricular pressure recording under basal condition or pharmacological stress. Myocardial fibrosis and molecular mediators of IR injury (reactive oxygen species, tumor necrosis factor-alpha and matrix-metalloproteinase-2) were assessed as well. Most of the rats subjected to IR did not show macroscopic signs of infarct, while only 17% of these animals showed large myocardial infarction scars. Of note, all animals submitted to IR presented the functional and molecular parameters altered when compared with the control subjects. Cardiac function was attenuated in all animals submitted to IR, regardless the presence or size of macroscopic cardiac scars. Interstitial fibrosis, matrix-metalloproteinase-2 activity and the expression of tumor necrosis factor-alpha were higher in the myocardium of all IR rats as compared to the control subjects (p<0.05). Myocardium superoxide anion and hydrogen peroxide were increased in rats without or with mild cardiac scars. These results show that IR leads to myocardial injury in rats. Besides, even the animals with an apparent healthy myocardium (without infarct scar) presented cardiac dysfunction and molecular changes that may contribute to the development of heart failure over time.

## Introduction

Reperfusion of the ischemic tissue is the most valuable therapeutic tool to limit the infarct area and preserve cardiac function after acute myocardial infarction (MI) [1,2]. However, despite the reperfusion therapy, a significant number of infarcted patients develop heart failure, approximately one year after MI [3,4]. Paradoxically, in some cases, reperfusion can worsen the ischemic scenario increasing the probability of life-threatening ventricular arrhythmias; besides, it also leads to myocardial damage through the death of the cardiomyocytes that were still viable during the ischemic period. This phenomenon is known as cardiac reperfusion injury; in essence, it may contribute to the final infarct size and, accordingly, to the long-term prognostic of the patient [5]. Therefore, the myocardial damage in a reperfused MI might be the result of two subsequent processes: ischemia and reperfusion.

Lack of oxygen and nutrient supply during ischemia result in biochemical and metabolic myocardial outcomes that may be aggravated by restoration of the aerobic metabolism provided by reperfusion [6,7]. Altered membrane potential, calcium overload, cell swelling, oxidative stress, mitochondrial permeability transition pore opening, activation of pro-inflammatory mediators and extracellular matrix proteases are the major outcomes that contribute for the reperfusion injury at the cellular level [7–9]. In this context, notwithstanding myocardial reperfusion has been demonstrated to be the best therapeutic option for acute MI, it is a “*double-edge sword*” [6]. Therefore, prevention of myocardial reperfusion injuries combined with the decrease of the incidence of heart failure, in patients surviving a reperfused MI, has been a remarkable challenge for physicians and scientists [6,8].

In this scenario, the study of experimental models of ischemia followed by reperfusion (IR) is crucial for improving the knowledge of the mechanisms involved in this situation. In most preclinical studies of experimental models of IR, short periods of myocardial reperfusion have been used [10]. Therefore, there is a lack of characterization of the long-term effects of IR in the literature. This drawback is fundamental for delineating future cardioprotective strategies to be translated into the clinical setting. Considering these facts, the present study was designed to characterize the long-term (4 weeks) repercussions of transitory (30 min) cardiac ischemia followed by reperfusion in contractile function as well as in morphological and molecular aspects of myocardium in rats.

## Materials and methods

### Ethics statement

Experiments were carried out in 65 male Wistar rats (supplied by the Animal Facility of the University of São Paulo, Campus of Ribeirão Preto, Brazil) submitted to myocardial IR or sham surgery (control group). The experimental protocol was reviewed and approved by the Committee of Ethics in Animal Research of the Ribeirão Preto Medical School, University of São Paulo, SP, Brazil (Protocol #163/2016). The personnel responsible for handling the animals in this study were adequately trained for this task.

### Experimental myocardial ischemia/reperfusion

Male Wistar rats (250–300 g) were anesthetized with ketamine and xylazine (50 and 10 mg/kg i.p., respectively), and received endotracheal intubation for mechanical ventilation with room air. A left thoracotomy was performed at the fifth intercostal space, to assess the heart, and the left anterior coronary artery (LAD) was temporarily (30 min) occluded with a polyester suture (4–0, Ethicon, Somerville, USA) placed between the pulmonary artery outflow tract and the left atrium. After 30 min of ischemia, the occlusion of the vessel was released by cutting the suture line. The thorax was immediately closed, and the excess air was removed from the chest cavity to prevent the development of pneumothorax. Sham rats (n = 7) were submitted to the same surgical procedures except for left coronary artery occlusion. The animals were led to recover from anesthesia on a heating pad (37°C) and received analgesic (tramadol hydrochloride: 2 mg/kg, during 3 consecutive days) and antibiotic (benzylpenicillin + streptomycin). After complete recovery from surgery, the rats were housed with free access to food and water on a temperature controlled (22±1°C) 12h light-dark environment. They were carefully checked every day after surgery to detect signs of suffering or distress.

### Experimental protocol

One month after IR, or sham surgery, the animals were anesthetized (urethane, 1 g/kg, i.p., Sigma-Aldrich, St. Louis, USA) and had their cardiac contractility evaluated by measuring the left ventricle (LV) pressure under baseline conditions and also during the infusion of increasing doses of dobutamine. Following, an additional dose of urethane (1g/kg, i.p., Sigma-Aldrich, St. Louis, USA) was administered for the animal euthanasia and subsequently tissue and blood sampling for histological and molecular analysis were collected immediately.

### Measurement of left ventricle systolic function during basal and stress conditions

Under the anesthesia of urethane (1 g/kg, i.p.), a catheter specially designed to measure ventricular pressure (SPR 320: Millar Instruments, Houston, TX, USA) was inserted into the LV via the right carotid. The catheter was connected, through a dedicated signal coupler (TC-510, Millar Instruments, USA), to a recording system (Bridge Amp attached to PowerLab/4SP, AD Instruments, Sydney, Australia) and, under continuous recording of LV pressure, they received increasing doses of dobutamine (1, 3, 10 and 15 μg/kg) intravenously. Dobutamine was used as a pharmacological stressor, and each dose was injected, in bolus, with an interval of at least 10 min between each other. The 1^st^ derivative in time of the LV pressure (dP/dt) was calculated online, and the maximum rate of increasing pressure was used as an index of systolic function of the rats.

### Tissue sampling

At the end of LV pressure recording, with the animals deeply anesthetized, they were euthanized, and the hearts were collected and cut transversely into two sections from the midventricular surface until the apex. The first midventricular section of each heart was fixed in phosphate-buffered 10% formalin and embedded in paraffin for histological analysis. The second section was rapidly frozen in liquid nitrogen and used for measuring matrix metalloproteinase-2 (MMP-2) activity, reactive oxygen species (ROS) and TNF-α gene expression.

### Assessment of MMP-2 activity by gelatin zymography

Samples were crushed in liquid nitrogen and homogenized overnight at 4°C in 10 mM Sodium Fluoride (#201154, Sigma-Aldrich, Saint Louis, USA), 1 mM Sodium Orthovanadate (#S6508, Sigma-Aldrich, Saint Louis, USA), 1X Protease Inhibitor Cocktail (#S8820, Sigma-Aldrich, USA) and cold RIPA buffer (#R0278, Sigma-Aldrich, Saint Louis, USA) according to the following proportion: 300 μL of buffer for 80 mg of sample. Homogenates were centrifuged with 12,000 rpm at 4°C for 20 min, and the supernatant was used. Extracts were then submitted to Bradford analysis (#B6916, Sigma-Aldrich, Saint Louis, USA) for protein quantification; 60 μg of the sample were loaded onto 8% polyacrylamide gels with 10% gelatin under non-reducing conditions. Fetal bovine serum was used as positive control. After electrophoresis, gels were incubated twice with 2.5% v/v Triton X-100 buffer for 30 min each at room temperature and then incubated with 50 mM Tris, 5 mM CaCl_2_.2H_2_O and 150 mM NaCl, pH 7.6 overnight at 37°C. Gels were coloured with 0.05% Coomassie Blue. The 75, 72 and 64 kDa matrix metalloproteinase-2 (MMP-2) were visualized using ChemiDoc Imaging Systems (Biorad, Hercules, USA) and quantified by the computer software ImageJ.

### Measurements of ROS generation in myocardial tissue: Lucigenin and amplex red assays

#### Lucigenin

Myocardial superoxide anion (O_2_^-^) generation was measured by a luminescence assay using lucigenin as the electron acceptor and NADPH as the substrate. Myocardial tissue from IR and sham rats were homogenized in an assay buffer (50 mM KH_2_PO_4_, 1 mM EGTA and 150 mM sucrose, pH 7.4) with a glass-to-glass homogenizer. The assay was performed with 100 μL of sample, lucigenin (5 μM), NADPH (0.1 mM) and assay buffer. Luminescence was measured for 30 cycles of 18 s each by a luminometer (Lumistar Galaxy, BMG Labtechnologies, Ortenberg, Germany). Basal readings were obtained prior to the addition of NADPH, and the reaction was started by the addition of the substrate. Basal and buffer blank values were subtracted from the NADPH-derived luminescence. O_2_^-^ was expressed as relative luminescence unit (RLU)/μg of protein.

#### Amplex red

Samples were frozen in Krebs, macerated and centrifuged. Fifty μL aliquots of the supernatant were removed and the amount of hydrogen peroxide (H_2_O_2_) produced by the myocardial tissue was determined fluorometrically by measuring the Amplex Red conversion (Molecular Probes, Invitrogen, Carlsbad, USA) (8x10^-6^ M) to a highly fluorescent compound resorufin, in the presence of horseradish peroxidase (4 U/mL). The resorufin fluorescence was detected by plate fluorimeter (Synergy 2 Multi-Detection Microplate Reader, BioTek Instruments) using excitation and emission wavelengths of 530 and 590 nm, respectively. The fluorescence values were corrected by the total amount of tissue proteins.

### TNF-α expression in myocardial tissue

For the analysis of Tumor Necrosis Factor-alpha (TNF-α) mRNA expression levels in the myocardium, total RNA was isolated from LV samples using Trizol (Invitrogen, Carlsbad, USA). RNA concentration and integrity were assessed. cDNA was synthesized using reverse transcriptase at 70°C for 10 min, followed by incubation at 42°C for 60 min and 75°C for 15 min. Taqman Gene Expression Assay for TNF-α (Rn01525859_g1) and the housekeeping gene Glyceraldehyde-3-phosphate dehydrogenase—GAPDH (Rn01775763_g1) were used. All samples were amplified in triplicates on StepOnePlus PCR System (Applied Biosystems, Foster City, USA) by using TaqMan Universal MasterMix (Applied Biosystems, Foster City, USA). PCR cycling conditions included 10 min at 95°C, followed by 40 cycles at 95°C for 15 s, 60°C for 1 min, and 72°C for 60 s. Dissociation curve analysis confirmed that signals corresponded to unique amplicons. TNF-α mRNA expression levels were normalized relatively to GAPDH mRNA levels using the comparative 2^-ΔΔCt^ method.

### Histological analysis and infarct size measurement

Midventricular cross-sections of the LV with 7 μm thick were cut and stained with picrosirius red for the quantification of interstitial collagen fibers. Stained cross-sections were captured using light microscopy (Leica DM5500B; Leica Microsystem, Wetzlar, Germany) at x40 magnification. To estimate the fraction area (%) of collagen in picrosirius red-stained sections, 15 images of the septum, for each rat, were randomly acquired for posterior analysis with the public-domain software NIH Image J. Infarct size was measured in picrosirius red-stained sections using Image J and calculated dividing the length of the infarcted area by the total circumference of the LV (expressed as percentage).

### Statistical analysis

Data are presented as means ± SEM. One-way ANOVA followed by the post hoc of Tukey was used to compare conventional echocardiographic parameters, tolerance to exercise, the maximum value of the positive dP/dt (+dP/dt_max) during basal conditions, molecular data and interstitial fibrosis. Two-way ANOVA followed by Holm-Sidak test was used to compare LV systolic function (+dP/dt_max), during dobutamine stress test, and segmental myocardial dysfunction by strain. The level of significance adopted was p<0.05.

## Results

### Mortality rates and classification of IR injuries

**Fig 1:**
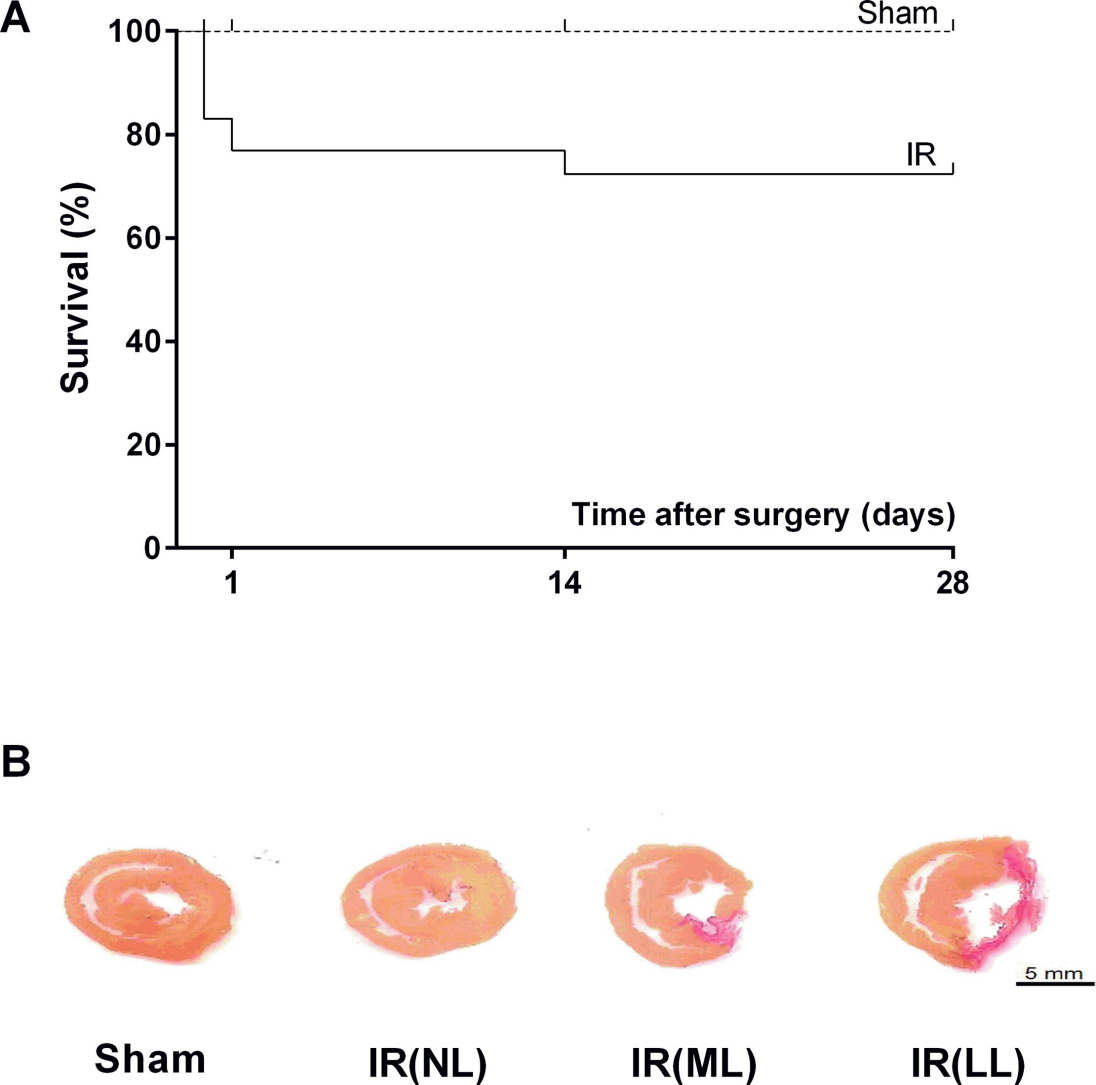
Survival rate and infarct scars in myocardial cross-sections. **(A)** Survival rate (Kaplan-Meier) from rats, following 4 weeks after sham-operation (Sham) or acute myocardial ischemia-reperfusion (IR). **(B)** Midventricular cross sections of the heart stained with *picrosirius red*. The animals were classified according to the presence and size of infarct scars. IR(NL): without a scar; IR(ML): mild infarct scars, i.e. less than 40% of the total circumference of the left ventricle; IR(LL): large infarct scars, i.e. more than 40% of the total circumference of the left ventricle.

At the onset of the reperfusion procedure, 11, out of 47, animals died. Also, 4 rats died within 24 hours after IR (post-operatory period), while only 3 animals died after 14 days after IR (Fig 1A). Considering all deaths, the mortality rate was 28% of all animals subjected to IR. All the deaths occurred suddenly, probably due to cardiac arrest caused by myocardial infarction/reperfusion (i.e., the animals were found dead in their cages). Euthanasia before the end of the experiments was not necessary since the rats did not exhibit signs of suffering or distress during the study. The survivors exhibited marked distinct sizes of macroscopic cardiac lesions (infarct scar), from no visible lesion up to large transmural scars spreading over the LV free wall. As indicated in Fig 1B, the animals were divided into 3 groups according to the size of cardiac scars: rats without macroscopic lesions (no lesion, NL), rats with infarcts scars not larger than 40% of the LV circumference (mild lesion, ML), and rats with transmural lesions that affected more than 40% of the circumference of the LV (large lesion, LL). The animals subjected to IR showed the following distribution: 45% of the animals did not show a cardiac lesion, 38% presented mild lesion while 17% exhibited a large infarct scar.

### General characteristics of rats with IR or sham surgery

The general characteristics observed in rats subjected to sham surgery or IR, with different degrees of cardiac lesions, are summarized in Table 1. Body weight was similar in all groups. Increased heart weight and heart/body weight ratio were observed in rats subjected to IR, denoting compensatory hypertrophy of the surviving myocardium. No differences were found in lung/body weight, and liver/body weight ratios among groups. These findings indicate no fluid accumulation in these organs. Accordingly, no sign of development of congestive heart failure was seen.

**Table 1:** General characteristics of rats 4 weeks after acute myocardial ischemia-reperfusion (IR) or sham surgery (Sham).

	Sham (n = 7)		IR(NL) (n = 6)		IR(ML) (n = 6)		IR(LL) (n = 6)	
Body weight (g)	419	± 0.01	485	± 0.03	567	± 0.01	547	± 0.02
Heart weight (mg)	1536	± 66	1853	± 113	2092	± 82	2600	± 231
Lung weight (mg)	1184	± 58	1492	± 175	2013	± 370	1778	± 113
Liver weight (mg)	14896	± 350	16740	± 638	19302	± 305	19398	± 1110
Heart/body weight (mg/g)	3.7	± 0.07	3.8	± 0.15	3.7	± 0.09	4.7	± 0.3*^+^^#^
Lung/body weight (mg/g)	2.8	± 0.13	3	± 0.3	3.5	± 0.6	3.2	± 0.2
Liver/body weight (mg/g)	36	± 1.7	35	± 1.5	34	± 0.7	35	± 1.2
Infarct size	0		0		21	± 3	46	± 1.2

NL: no lesion, ML: mild lesion and LL: large lesion.

* P<0.05 vs. Sham

+ P<0.05 vs. IR (NL)

# P<0.05 vs. IR (ML). Values are means ± SEM

### Cardiac function analysis and the pharmacological stress test

**Fig 2:**
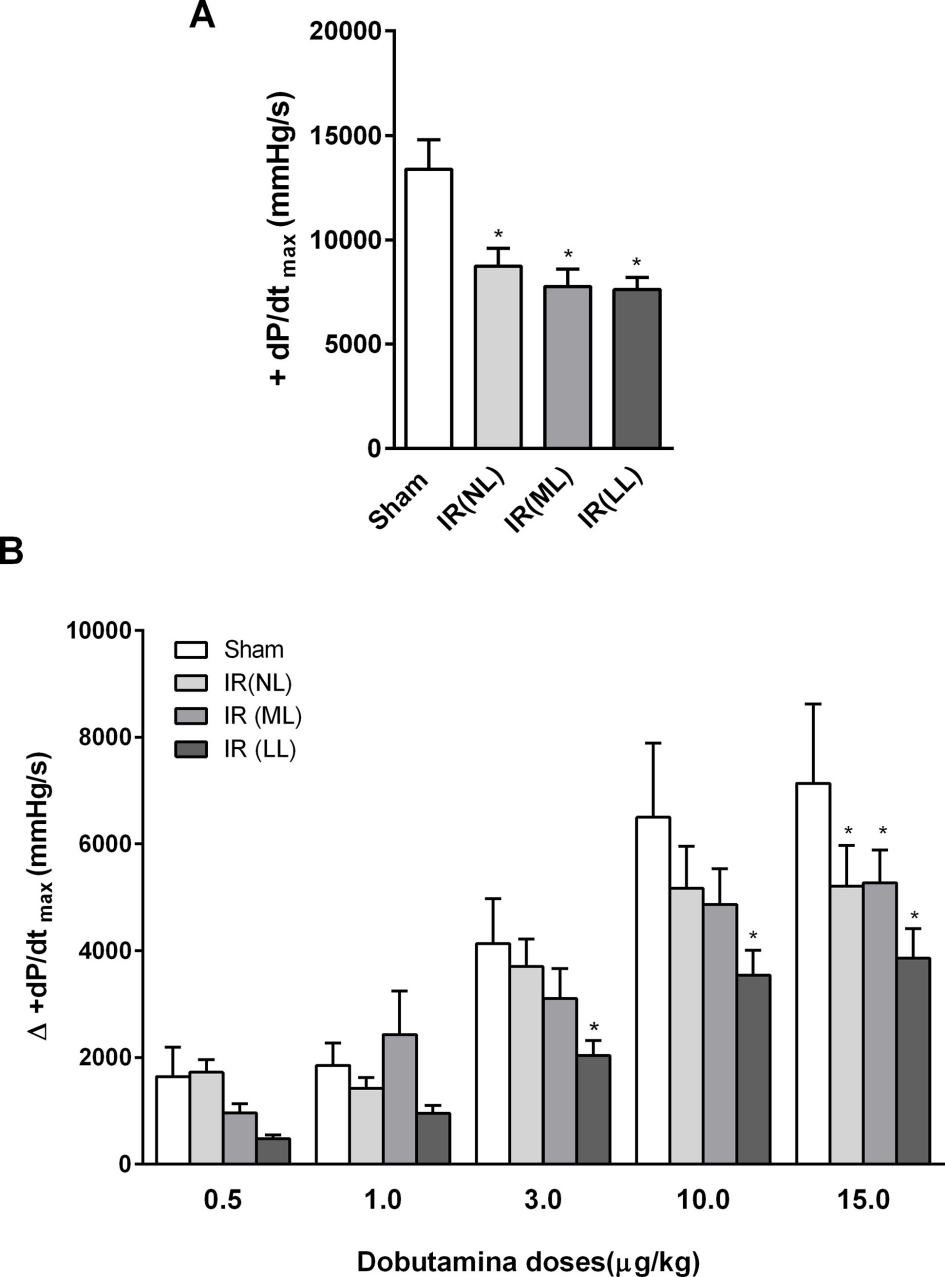
Cardiac function in basal and stress conditions. **(A)** Basal recordings of maximal increasing rate of left ventricular pressure (+dP/dt_max) and **(B)** Changes in +dP/dt_max elicited by 1, 3, 10 and 15 μm/kg of dobutamine infusion from rats following 4 weeks after sham-operation (Sham) or acute myocardial ischemia-reperfusion (IR), with no (NL), mild (ML) or large (LL) cardiac lesions. Values are means ± SEM, * vs. Sham, P<0.05.

The maximum increasing rate of LV pressure measured by the maximum value of the positive dP/dt (+dP/dt_max), as well as dP/dt response to increasing doses of dobutamine, is shown in Fig 2. Note that basal +dP/dt_max was found smaller in rats subjected to IR, independently of the presence of the myocardial scar, as compared to sham-operated counterparts. Dobutamine elicited, as expected, a dose-dependent increase in +dP/dt_max in all rats. IR rats with large cardiac lesions presented impaired response of +dP/dt_max to 3, 10 and 15 μg/kg of dobutamine as compared to their sham-operated counterparts. Nevertheless, at the dose of 15 μg/kg, dobutamine elicited an impaired response of +dP/dt_max in all rats subjected to IR, i.e. independent of the presence of macroscopic cardiac lesions.

### Myocardial ROS generation and TNF-α expression

**Fig 3:**
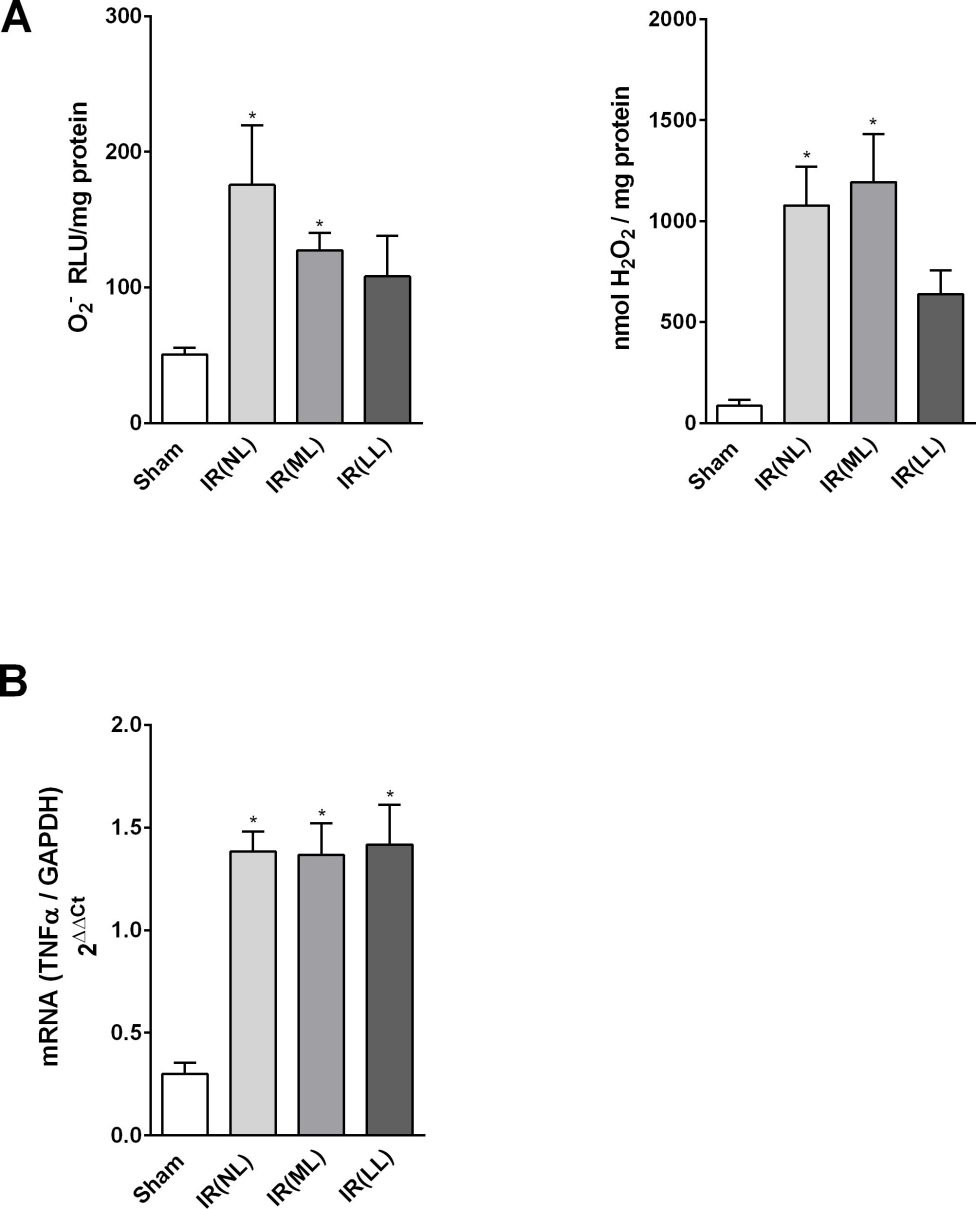
Myocardial ROS and TNF-α expression. **(A)** Myocardial tissue concentrations of O_2_^-^ and H_2_O_2_ and **(B)** TNF-α mRNA expression in rats 4 weeks after sham-operation (Sham) or acute myocardial ischemia-reperfusion (IR), with no (NL), mild (ML) or large (LL) cardiac lesions. Values are means ± SEM, *vs. Sham, P<0.05.

Data on O_2_^-^ and H_2_O_2_ generation, and TNF-α gene expression are shown in Fig 3. High concentrations of O_2_^-^ and H_2_O_2_ were found in the IR rats with no or mild cardiac lesions as compared to sham-operated rats, whereas in IR rats with large myocardial scars the slight increase of O_2_^-^ and H_2_O_2_ was not statistically significant. TNF-α mRNA expression was high in all animals submitted to IR despite the presence, or absence, of infarction scar.

### Interstitial collagen deposition and myocardial MMP-2 activity

**Fig 4:**
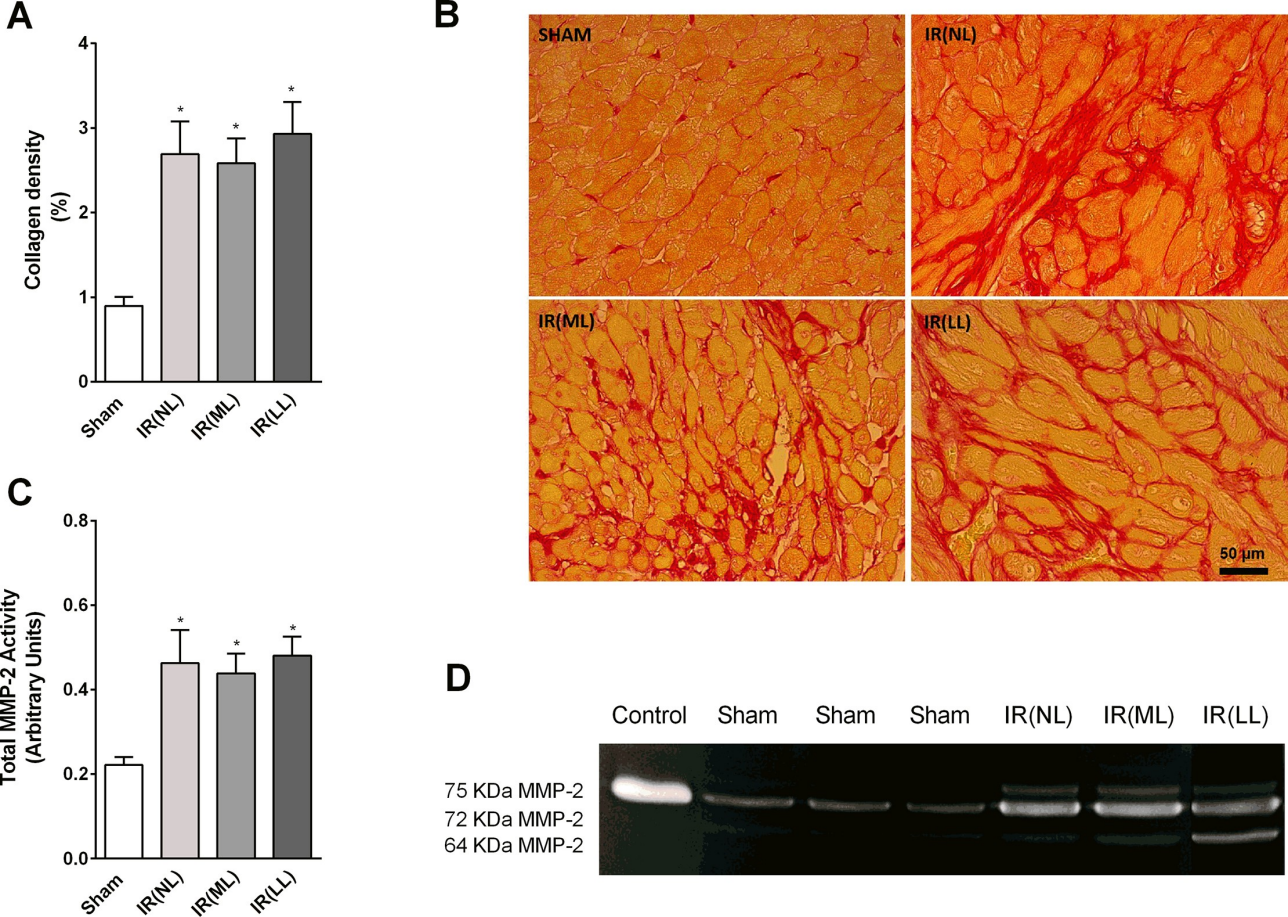
Myocardial fibrosis and MMP-2 activity. **(A)** Quantification of septal collagen density and **(B)** photomicrographs of myocardial tissue stained with picrosirius red in rats, 4 weeks after sham-operation (Sham) or acute myocardial ischemia-reperfusion (IR), with no (NL), mild (ML) or large (LL) cardiac lesions. **(C)** Quantified values of MMP-2 activity and **(D)** its respective gelatin zymogram. Values are means ± SEM, * vs. Sham, P<0.05.

The density of septal collagen (Fig 4A) was greater in all rats subjected to IR, independently of the degree of cardiac lesion. Also, all rats subjected to IR showed increased total MMP-2 activity (Fig 4C) when compared to sham-operated rats, thus indicating that the repairing process may be present in noninfarcted areas as well.

## Discussion

Despite the surgery to induce IR in all animals has been performed by the same researcher, following strictly the same procedures, a marked heterogeneity was observed in macroscopic injury among the animals. This finding was actually expected and interpreted mainly as a consequence of anatomical variations of coronary vessels. Moreover, the present study demonstrates, as expected, that myocardial IR elicited different degrees of cardiac injury in rats. However, this study shows, for the first time, that even the animals with an apparently healthy myocardium (without infarct scar) presented LV systolic dysfunction, under a basal and challenged condition, as well as marked changes in cardiac histology (fibrosis) and molecular mediators involved in cardiac dysfunction and myocardial remodeling.

After 30 min of ischemia followed by 4 weeks of reperfusion, only a small fraction (17%) of the surviving rats presented large transmural MI scar combined with severe systolic dysfunction. Another fraction (38%) of survivors from IR displayed moderate infarct leading to scars that measured less than 40% of the total area of the LV. These animals showed a significant reduction in systolic function during basal and stress conditions, but not as severe as their counterparts with large cardiac scars.

Although the majority of the surviving animals (45%) did not show any macroscopic sign of myocardial infarct (Fig 1), surprisingly, these animals showed impairment of LV systolic function and higher septal accumulation of interstitial collagen combined with high total MMP-2 activity. Furthermore, elevated concentrations of ROS (O_2_^-^ and H_2_O_2_) and higher levels of TNF- α were detected in the myocardial tissue from rats subjected to IR without cardiac scars.

During reperfusion, a burst of ROS is produced by a variety of sources, especially by the disruption of mitochondrial membrane potential [8,11,12]. ROS accumulation stimulates pro-inflammatory pathways and causes direct damage to the cellular DNA, proteins and lipids [11]. Therefore, redox-sensitive transcription factors such as nuclear factor-κB were activated by ROS eliciting the expression of TNF-α and other pro-inflammatory cytokines [13,14]; this mechanism plays an important role in the impairment of calcium homeostasis and consequently in myocardial contractile dysfunction [15]. In addition, it has been postulated that ROS contributes to the activation of MMP-2 performing a covalent modification of its Cys residue between the pro-peptide and the catalytic domain [16].

MMP-2, also known as gelatinase A or type IV collagenase, plays a critical role in cardiovascular diseases [17] and has emerged as a key enzyme involved in cardiac conditions associated with increased oxidative stress [16–20]. Moreover, MMP-2 was found increased in the coronary effluent, immediately after IR of isolated rat hearts [21]. MMP-2 has been considered the major protease involved in myocardial stunning [19,22]. Beyond its action in the extracellular matrix, MMP-2 modulates different cellular functions during the IR process, for instance, platelet aggregation [23], vascular tone [24,25] and digestion of troponin I [19,22,26] and myosin light chain 1 [27], leading to cardiac contractile dysfunction.

These outcomes might be involved with the impairment of myocardial function observed after IR. Therefore, even in the animals without macroscopic cardiac lesions, the IR triggers molecular mechanisms that culminate with adverse changes in the ventricular architecture and contractile dysfunction under stress; accordingly, determining increased ventricular stiffness over time.

Taking into account these data, the present study provides novel information regarding long-term outcomes after myocardial IR in rats. The important and novel information brought by this study is that, despite the degree of a macroscopic myocardial lesion, all animals presented cardiac dysfunction with a certain degree of severity. Moreover, our findings revealed that, all survivors from myocardial IR, even those without any visible infarction scar, displayed interstitial fibrosis and derangements at the molecular level; outcomes, probably involved in the development of heart failure over time. Studies with dogs and humans suggested that myocardium at risk, collateral flow and metabolic demand are determinant factors for infarct size after reperfusion [28,29]; however, future studies are required to elucidate the mechanism(s) involved in molecular alterations observed in IR animals without patent infarct scar.

Also, it is important to emphasize that a consistent characterization of animal models with different methodological protocols are crucial; because these models may remarkably contribute to improving therapeutic strategies in the field of IR.

## Supporting information

S1 File Experimental dataset. Fig A. Cardiac stress test; Fig B. Superoxide anion; Fig C. Hydrogen peroxide; Fig D. TNF alpha; Fig E. Collagen density; Fig F. MMP2.
(XLSX)

S2 File Septal collagen deposition. Midventricular cross-sections of the left ventricle stained with picrosirius red for the quantification of interstitial collagen fibers.
(XLSX)
